# The impact of walking on creative thinking: A systematic review and meta-analysis

**DOI:** 10.1371/journal.pone.0347878

**Published:** 2026-05-13

**Authors:** Alex Thabane, Vikram Arora, Joelle Boilard, Adam Sutoski, Sameer Parpia, Goran Calic, Jason W. Busse, Tyler McKechnie, Phillip Staibano, Mark Phillips, Roni Reiter-Palmon, Mohit Bhandari

**Affiliations:** 1 Department of Health Research Methods, Evidence, and Impact, McMaster University, Hamilton, Canada; 2 Temerty Faculty of Medicine, University of Toronto, Toronto, Ontario, Canada; 3 Department of Physiology, University of Alberta, Edmonton, Canada; 4 Department of Oncology, McMaster University, Hamilton, Canada; 5 DeGroote School of Business, McMaster University, Hamilton, Canada; 6 Department of Anesthesia, McMaster University, Hamilton, Canada; 7 Department of Surgery, McMaster University, Hamilton, Canada; 8 Department of Psychology, University of Nebraska at Omaha, Omaha, United States of America; Grigore T Popa University of Medicine and Pharmacy Iasi: Universitatea de Medicina si Farmacie Grigore T Popa lasi, ROMANIA

## Abstract

**Background:**

Walking is associated with many benefits, from improved mental health to a reduced risk of mortality – but can it boost creative thinking? Current evidence suggests a positive effect of physical activity on creative thinking, but the specific effect of walking has not been adequately explored.

**Methods:**

We conducted a systematic search of the PsycINFO, MEDLINE, Scopus, and ProQuest databases from inception to 8 January 2025 using search terms related to walking and creativity. We then performed meta-analyses of Cohen’s *d* effect sizes to assess the effect of walking on creative thinking, as represented by divergent thinking (the ability to generate novel and useful ideas) and convergent thinking (the ability to analyze and select ideas to find the best solution). We assessed study quality using a creativity-specific tool, and the certainty of the evidence with the Grading of Recommendations Assessment, Development and Evaluation Framework.

**Results:**

We identified 23 studies (12 randomized experimental studies, nine non-randomized experimental studies, two observational studies) from 16 articles including a total of 1,036 participants, most of whom were post-secondary students. We found moderate certainty evidence of a large effect of walking on divergent thinking (*d* = 0.93 [95% CI 0.44, 1.42]), and very uncertain evidence of a null effect of walking on convergent thinking (*d* = 0.16 [95% CI −0.31, 0.63]). Sensitivity analyses of randomized trials only found similarly large effects of walking on divergent thinking ability (*d* = 0.82 [95% CI 0.35, 1.28]).

**Conclusion:**

Results suggest that walking likely results in a large increase in divergent thinking, indicating its potential as an intervention to stimulate creative idea generation.

## Introduction

“*All truly great thoughts are conceived while walking*.”

Friedrich Nietzsche

Despite its physiological complexity, walking is remarkably simple for humans. It appears to be instinctual: fetuses have been observed performing the same alternating pattern of leg movements characteristic of walking [1,2]. In addition to being the primary mode of human mobility, walking is associated with a range of benefits including improved cardiovascular and mental health [3,4], better memory and executive function [5,6], and a reduced risk of mortality [7]. But its benefits may extend further: many great thinkers – from Albert Einstein to Friedrich Nietzsche – have lauded the ability of walking to stimulate the imagination and thought. This begs the question: could it be possible to walk into more creative ideas?

Creativity can be defined as interaction between aptitude, process, and environment leading to the production of novel and useful ideas or products [8]. It is a critical 21^st^ century ability, with both intrinsic and instrumental value to society – beyond being personally meaningful to the creator, creative ideas can solve important problems and result in innovative products that garner rewards, recognition, and financial remuneration [9]. Naturally, there is interest from scientific and lay communities in interventions and activities that can improve one’s ability to think creatively.

Formal creativity training has shown effectiveness in improving creative thinking [10]; this importantly illustrates the plasticity of human creativity. However, interventions and activities capable of enhancing human creativity are not limited to deliberate creativity training. Two previous systematic reviews found that physical activity, such as cycling, dance, running, and other aerobic activities, can enhance creative thinking [11,12]; similar findings have also been reported in child samples [13]. These findings make physical activity a promising lever for improving our ability to think creatively.

While the aforementioned reviews included some studies on walking, they did not empirically explore the differences in the effect of physical activity on creative thinking by activity type. Given that higher-intensity exercise has been found to result in differential effects compared to walking in cognitive function and mental health outcomes [14], both of which are associated with creativity [15,16], it’s possible and likely that similar differences in the effect on creative thinking might exist. More, some reviews focused on divergent thinking (i.e., the ability to generate novel and useful ideas), but left out convergent thinking (i.e., the ability to analyze and select ideas to find the best solution), which is a critical aspect of effective creative thinking [17–19]. It follows that a meta-analytic assessment of the effect of walking on both divergent and convergent thinking was warranted. Given that walking is an accessible activity for people of all ages, such evidence would have broader applicability than sports and other high-intensity physical activities.

We did find a narrative review that explored the impact of physical activity on creative thinking by activity type, including the specific effects of walking [20]. While this review provided some indication of the specific benefits of walking on creative thinking, it was limited by the lack of study quality assessment and meta-analytic synthesis, and a limited database search which led to several relevant studies being omitted. To fill this gap in the literature, we conducted a comprehensive systematic review and meta-analyses with the primary objective of assessing the effect of walking on creative thinking, as measured by both divergent and convergent thinking. As a secondary objective, we explored whether the effect of walking differs depending on if It occurs outdoors vs. indoors.

Considering the previous literature [11,20–24], we hypothesized that walking would have a significant effect on divergent thinking but not convergent thinking, and that outdoor walking would have a greater effect than indoor walking.

## Methods

This paper was reported in accordance with the Preferred Reporting Items for Systematic reviews and Meta-Analyses (PRISMA) guidance for the reporting of systematic reviews (S1 Appendix) [25] and registered in PROSPERO (CRD420251079468). Ethics approval was not required to conduct this study. The data and code from this study can be found through the Harvard Dataverse [26].

### Eligibility criteria

We set out to identify articles reporting primary research conducted in an adult human population (>18 or older; or in a post-secondary institution or higher), studying the effect of walking on the outcome of creative thinking (as represented by divergent thinking and convergent thinking [27]). Common measures of divergent thinking included the Alternate Uses Task (AUT) and Torrance Test of Creative Thinking (TTCT); common measures of convergent thinking include the Remote Associates Task (RAT). We excluded studies that explored the use of stair climbing as the exposure/intervention, as well as studies that measured verbal fluency as the sole measure of creativity, as it does not account for novelty which is a requisite of creativity [8,28].

In the case that multiple experiments were included in the same article, all experiments were individually assessed for inclusion, with the prerequisite that the samples across experiments were independent and thus no correlation in results across experiments was expected. In the case that the same sample was used in multiple experiments, we included the experiment with the largest sample size and/or the experiment that utilized a measure of creative thinking with established or reported reliability and validity data. We considered both as dissertations and peer-reviewed journal articles as eligible; if a dissertation was later published in a journal, we included the journal article. We considered all articles regardless of the language of publication; in the event an eligible non-English article was identified, we planned to convert it into English using a translation software.

### Search strategy and information sources

We conducted literature searches the following databases from inception to 8 January 2025: PsycINFO, MEDLINE (PubMed), Scopus, and ProQuest Dissertations. We developed tailored search strategies for each database using key words and subject headings for the concepts of walking and creativity, combined with the Boolean operators AND/OR. The complete search strategy can be found in S2 Appendix.

Additionally, we hand-searched the references of two systematic reviews exploring the effect of physical activity on creativity [11,20], performed a Google Scholar search using the keywords ‘creativity’, ‘divergent thinking’, ‘convergent thinking’, and ‘walking’, and manually searched the reference of any articles which were ultimately included in this review.

### Study selection process

The results of the database searches and manual searches of systematic review references were imported into Covidence software [29] for the study selection process. In the first phase of the study selection process, we screened eligible studies based on their titles and abstracts. Both title and abstract screening and full-text review were performed independently and in duplicate by a team of three reviewers (VA; JB; AS); any discrepancies were resolved by discussion until consensus, or adjudication by the lead author (AT) if necessary.

### Data extraction

Two individuals (JB; VA) independently extracted the following data from the included studies: year of publication; study design; first author; journal; sample characteristics; description of walking intervention/exposure; description of control intervention/exposure; name and type of creative thinking assessment(s); and creativity testing results. Any discrepancies were resolved by discussion between the two extractors until a consensus was reached. All consensus data was cross-checked by the lead author (AT) to ensure its accuracy.

### Outcome(s)

Our primary outcome of interest was creative thinking, as constituted by divergent thinking (i.e., the ability to generate novel and useful ideas) and convergent thinking (i.e., the ability to analyze and select ideas to find the correct or best solution).

### Study quality assessment

To assess the quality of the included studies, we used a risk of bias scale consisting of 8 items, developed Frith and colleagues [30] specifically for creativity research studies (S3 Appendix), based on the Cochrane Risk of Bias Tool [31]. We conducted assessments independently and in duplicate (AT; VA), with discrepancies resolved by discussion until a consensus was reached. The sum of all 8 item scores was considered the total study quality score; studies with scores between 6–8 were at low risk of bias; 3–5 at moderate risk of bias; and 0–2 at high risk of bias. For items 2 and 3, most studies did not report evidence of validity or reliability: we therefore conducted our own database searches for reliability and validity data using Google Scholar, PsycINFO, and MEDLINE, as we felt the penalization of common and generally accepted tests of creative thinking (e.g., the AUT) during study quality assessment due to lack of reporting was inappropriate. For item 4, we considered creativity test evaluation by multiple reviewers, Artificial Intelligence (AI)-based scoring techniques, or tests with a single correct response (e.g., Remote Associates Task) to be scoring procedures robust to bias.

To assess the certainty of the evidence, we used the Grading of Recommendations Assessment, Development and Evaluation (GRADE) system [32], assessing each outcome across five domains (i.e., risk of bias, inconsistency, indirectness, imprecision, publication bias) and reporting the results in tables.

### Statistical analyses

We performed random effects meta-analyses of Cohen’s *d* effect sizes for both divergent and convergent thinking. Cohen’s *d* was chosen as the effect measure of choice as it is highly prevalent in psychology research and has been used in many existing meta-analyses in the field of creativity [33–37]. We used the reported Cohen’s *d* effect size from each study, or calculated it using the reported means, standard deviation(s), and sample sizes. In the event such data were not reported, we transformed any *t*-test statistics and *F*-test statistics representing the effect of walking on creative thinking into Cohen’s *d* effect sizes using the function *esc* in R Software [38]. We assessed heterogeneity using the *I*^2^ and τ^2^ (tau-squared) statistics and, when more than 10 studies were included in a meta-analysis, assessed the risk of publication bias through funnel plot inspection and the Egger’s regression test.

For our secondary objective, we performed a sub-group analysis of the effect of walking on divergent thinking by walking location (i.e., indoor walking; outdoor walking). We also performed two *post-hoc* subgroup analyses to explore whether the effect of walking differed depending on the study design (i.e., within-subjects; between-subjects) and type of creativity test administered.

Finally, we performed a sensitivity analysis of the effect of walking and divergent thinking including only studies using a randomized design, which have improved internal validity due to lower risk of selection bias and the balancing of known and unknown confounding variables [39,40].

We made several decisions during data extraction to facilitate the conduct of a valid meta-analysis, which we report here for transparency. Where multiple types of walking interventions were studied in the same experiment, we used data from the intervention that most closely represented walking in natural conditions (e.g., free walking as opposed to constrained walking or walking in a rectangular path). We also to opted to utilize data on outdoor walking instead of indoor walking in the case that both conditions were studied in the same experiment and only one control group was used. These conditions were chosen as they best represent how walking is most naturally experienced in a real-world setting. When creative thinking was assessed both during and after walking, we used the after walking test scores. With respect to indices of divergent thinking, we used the novelty/originality scores from the respective tests; when such data was not reported, we used flexibility scores.

Using effect size rules of thumb by Sawilowsky (2009), we interpreted pooled Cohen’s *d* effect sizes to be either small (*d* = 0.20–0.49), medium (*d* = 0.50–0.79), large (*d* = 0.8–1.19), very large (*d* = 1.20–1.99) and huge (*d* = 2.00+) [41]. All analyses were performed in R software (version 4.3.2) via R Studio using the *metagen* and *forest* packages [42]. The results of all meta-analyses were presented in forest plots. An α-level of 0.05 was considered the threshold for statistical significance, with no adjustments for multiple testing.

## Results

### Literature search

**Fig 1:**
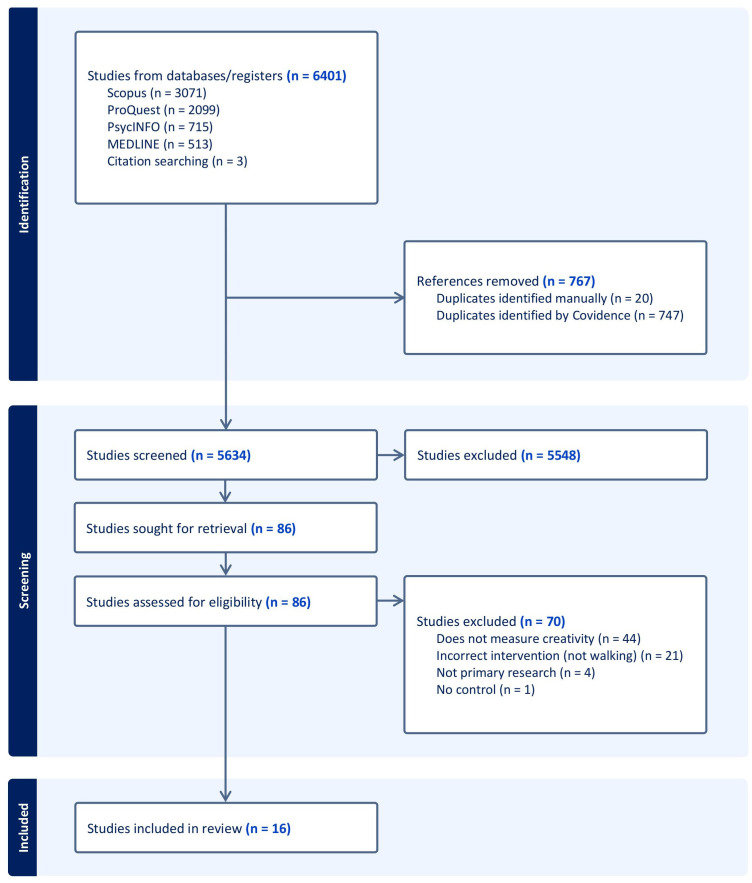
PRISMA Flow Diagram.

After removing 767 duplicates, our literature search identified 5,634 unique citations. Of those, 86 underwent full-text review; 16 articles, comprised of 23 individual studies, were ultimately included in this systematic review and meta-analysis [23,43–57]. The completed details of the literature search process are reported in **Fig 1**.

### Study characteristics

A total of 1,036 participants were included across the 23 studies, most of which were post-secondary students. Twelve studies utilized a randomized experimental design, nine studies a non-randomized experimental design, and two studies an observational design. The majority of studies experimentally studied walking in an indoor setting (n = 17; 73.9%), measured creative thinking with the AUT (n = 16; 69.6%), and were published in the last 10 years (n = 15; 65.2%). Seven (30.4%) studies assessed convergent thinking, mostly using a version of the RAT. We found most studies were of low risk of bias. A full description of the included study characteristics and risk of bias assessments can be found in **Table 1** and S4 Appendix, respectively.

**Table 1:** Study Characteristics.

Study	Design	Participants	Age	Walking Group	Control Group	Creativity Task(s)	RoB Score (/8)
Between-Subject Designs							
Bartholomae 2024 [44]	Randomized controlled trial	49 university students	18-20	Treadmill walking (2.5 mph)	Standing on treadmill	AUT	7
Leung 2012 (Study 2b) [46]	Randomized controlled trial	104 post-secondary students	NR	2-minute indoor free walking	Seated	Droodle Task Lego Task	5
Main 2018 (Experiment 1) [47]	Randomized controlled trial	29 undergraduate students	20.4	8-minute treadmill walking	Seated	AUT	7
Main 2018 (Experiment 2) [47]	Randomized controlled trial	56 undergraduate students	23.5	8-minute outdoor walking (*n* = 22) 8-minute treadmill walking (*n* = 17)	Seated (*n* = 17)	AUT	8
Oppezzo 2012 (Experiment 1) [49]	Randomized controlled trial	40 participants	NR	Outdoor walking along a path	Seated inside	AUT	7
Oppezzo 2012 (Experiment 2) [49]	Randomized controlled trial	40 participants	NR	Walking indoors (*n* = 10) Walking outdoors (*n* = 10)	Seated indoors (*n* = 10) Seated outdoors (*n* = 10)	Barron’s Symbolic Equivalence Test	6
Oppezzo 2012 (Experiment 3) [49]	Randomized controlled trial	48 college students	NR	Treadmill walking	Seated	AUT	7
Oppezzo 2014 (Experiment 2) [50]	Randomized controlled trial	48 community college students	NR	Self-paced treadmill walking (*n* = 16)	Seated (*n* = 32)	AUT	8
Oppezzo 2014 (Experiment 3) [50]	Non-randomized trial	40 university students	NR	Outdoor walking along a path (*n* = 20)	Seated (*n* = 20)	AUT	7
Palmer 1995 [51]	Randomized controlled trial	44 participants	66.4 (4.2)	16 weeks progressive walking protocol (*n* = 16)	Strength and flexibility exercise protocol (*n* = 15) No changes in their lifestyle, diet, or exercise (*n* = 13)	AUT	7
Within-Subject Designs							
Abdullah 2016 [43]	Non-randomized trial	21 participants	NR	20 minutes of outdoor walking for 3 days	**—**	AUT Compound-RAT	7
Frith 2018 [55]	Non-randomized trial (counter-balanced)	32 university students	23.1 (3.4)	15-minute treadmill walking (≥3.0 mph)	**—**	AUT RAT	7
Frith 2021 [57]	Non-randomized trial (counter-balanced)	32 university students	22.7 (3.23)	15-minute treadmill walking	**—**	Instances Creativity Task	6
Frith 2022 [56]	Non-randomized (counter-balanced)	45 participants	21.1 (1.9)	15-minute treadmill walking	**—**	RAT	8
Jung 2023 [45]	Non-randomized (counter-balanced)	20 undergraduate students	21.2 (1.0)	20-minute treadmill walking	**—**	Insight Creativity Task	6
Murali 2022 (Experiment 1) [48]	Randomized controlled trial	20 undergraduate students	18-35	4-minute indoor free walking	**—**	AUT	7
Murali 2022 (Experiment 2) [48]	Randomized controlled trial	17 participants	18-35	4-minute indoor free walking	**—**	AUT	7
Murali 2022 (Experiment 3) [48]	Randomized controlled trial	23 participants	18-35	3.75-minute indoor free walking	**—**	AUT	7
Oppezzo 2014 (Experiment 1)	Non-randomized trial	48 undergraduate students	NR	Self-paced treadmill walking	**—**	AUT RAT	7
Patterson 2018 [52]	Non-randomized trial	20 college students	21.4 (1.3)	15-minute self-paced treadmill walking	**—**	AUT Compound-RAT	7
Zhou 2017 (Experiment 1B) [54]	Non-randomized (counter-balanced)	63 college students	21.3 (range: 18–25)	Indoor free roaming	**—**	Consequences Imagination Task	6
Observational Designs							
Rominger 2024 [53]	Cross-sectional	157 university students	23.3 (3.7)	Number of steps over 5-day period via accelerometer	**—**	Verbal and Figural Ambulatory Battery of Creativity	4
Chen 2021 [23]	Cross-sectional	40 undergraduate students	23.0 (2.0)	Self-reported frequency of walking	**—**	AUT Matchstick arithmetic problems	3

*RoB = risk of bias, assessed with the Frith (2019) study quality scale.*

*AUT = Guilford’s Alternate Uses Task.*

*RAT = Remote Associates Task.*

*NR = Not Reported.*

*For between-subject study experiments, some studies did not report individual group sample sizes.*

### Divergent thinking

**Fig 2:**
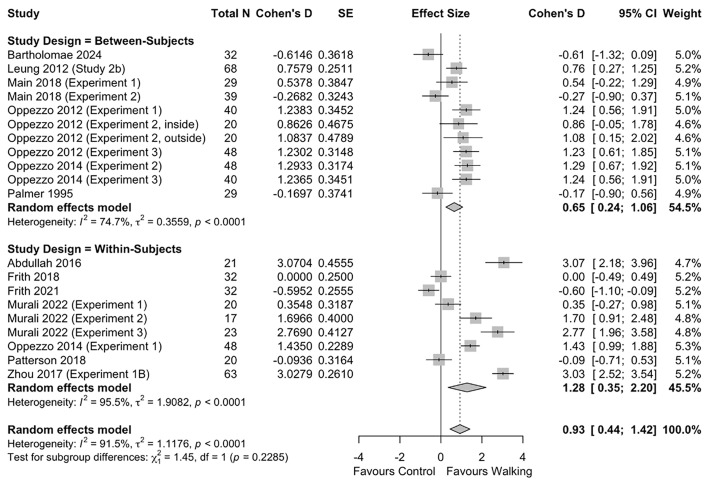
Effect of walking on divergent thinking.

Twenty studies were included in the meta-analysis of the effect of walking on divergent thinking, 11 using a between-subjects design and nine using a within-subjects design. Meta-analysis results revealed a large effect of walking on divergent thinking (*d* = 0.93 [95% CI 0.44, 1.42], p < 0.001) (**Fig 2**). In the *post-hoc* sub-group analysis by study design (i.e., between-subjects vs. within-subjects), we found no difference in effect size between the two designs (*p* = 0.229) (**Fig 2**).

**Fig 3:**
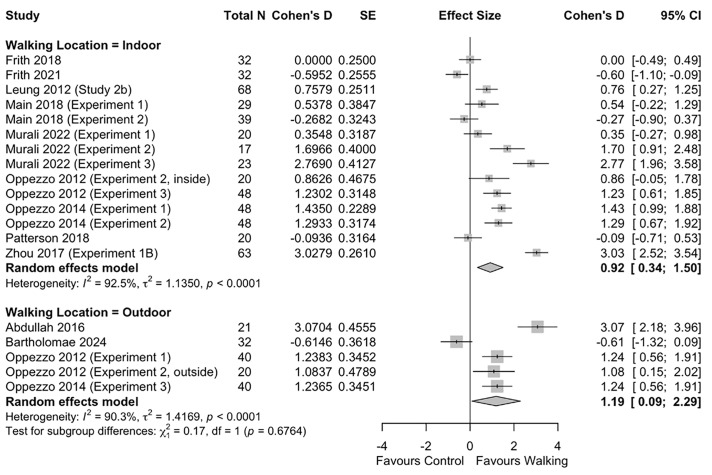
Sub-group analysis of effect of walking on divergent thinking by walking location.

In the sub-group analysis by walking location, we found no significant difference in the effect of walking when conducted indoors vs. outdoors (*p* = 0.676) (**Fig 3**).

**Fig 4:**
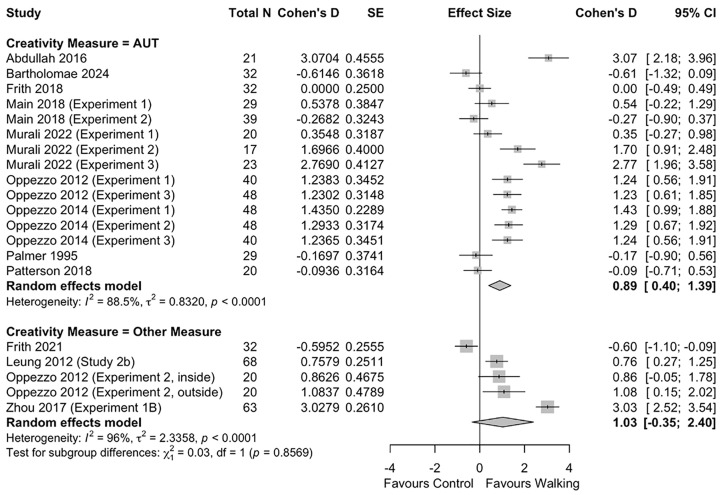
Sub-group analysis of effect of walking on divergent thinking by creativity measure.

Similarly, the *post-hoc* sub-group analysis by creativity assessment method found no difference in effect size between studies using the AUT and those using another measure of divergent thinking (*p* = 0.857) (**Fig 4**).

**Fig 5:**
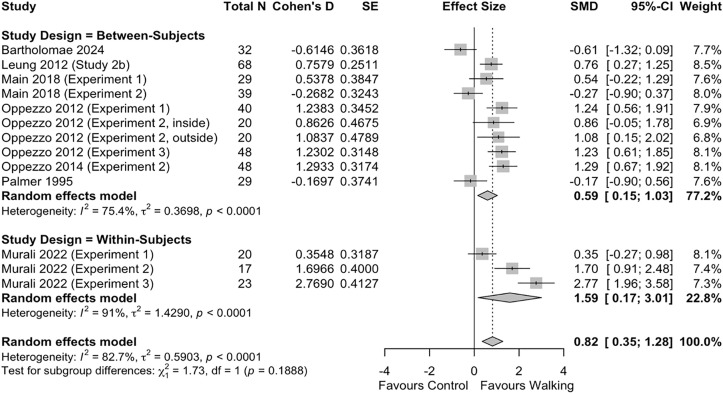
Sensitivity analysis of effect of walking on divergent thinking, randomized studies only.

In the sensitivity analyses of only randomized studies, a large effect of walking on divergent thinking was observed (*d* = 0.82 [95% CI 0.35, 1.28]) (**Fig 5**), which was comparable in magnitude and interpretation to that of the primary analysis.

### Convergent thinking

**Fig 6:**
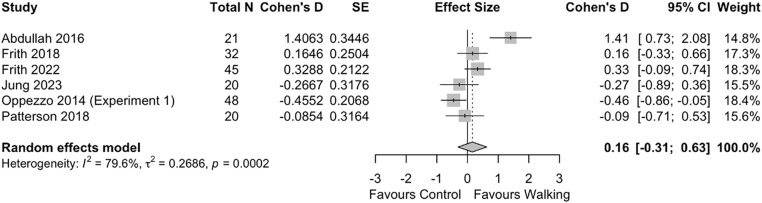
Effect of walking on convergent thinking.

Six studies were included in this meta-analysis of the effect of walking on convergent thinking, all utilizing a within-subjects study design. The meta-analysis results found no significant effect of walking on convergent thinking ability (*d* = 0.16 [95% CI −0.31, 0.63]) (**Fig 6**). Sub-group and sensitivity analyses were not possible due to the limited number of studies.

### Observational studies

We found two cross-sectional studies assessing the relationship between walking and creative thinking [23,53]. In Rominger 2024, which sampled 157 young adults, both acute bouts of walking and regular walking were associated with greater divergent thinking ability [53]. In Chen 2021, which assessed the cross-sectional data from a previous randomized trial, self-reported walking was associated with more original ideas on the AUT, but had no association with convergent thinking [23].

### Publication bias

**Fig 7:**
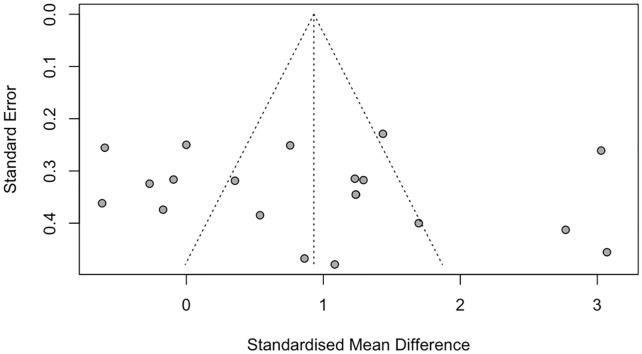
Funnel Plot for Divergent Thinking.

We found no evidence of publication bias in the outcome of divergent thinking, as assessed by visual funnel plot inspection (**Fig 7**) and the Egger’s regression test (p = 0.586).

### GRADE assessment

We rated the quality of evidence for the outcome of divergent thinking as ‘moderate’ based on the sensitivity analyses of randomized studies, downrating for inconsistency (**Table 2**). We rated the evidence on convergent thinking as ‘very low’, rating down for study type, inconsistency, and imprecision.

**Table 2:** GRADE Evidence Profile.

Outcome	Studies (*n*)	Certainty Assessment					Relative Effect Size
		*Risk of Bias*	*Inconsistency*	*Indirectness*	*Imprecision*	*Publication Bias*	
Divergent Thinking^1^	13 *(433)*	No limitations	Some limitations^2^	No limitations	No limitations	No limitations	Moderate
							Pooled Cohen’s *d* = 0.82 [0.35, 1.28]
Convergent Thinking^3^	6 *(186)*	No limitations	Some limitations^4^	No limitations	No limitations^3^	No limitations	Very low
							Pooled Cohen’s *d* = 0.16 [−0.31, 0.63]

^1^ = we report here the GRADE for the sensitivity analyses of randomized trials only, as the certainty of evidence was higher than the primary analyses with a similar point estimate and interpretation (i.e., large effect)

^2^ = we rated down once due to the high heterogeneity in effect estimates across included studies in the sensitivity analyses (*I*^2^ = 82.7%)

^3^ = we rated down twice for study design, as all included studies were observational in nature

^4^ = we did not rate down the same effect estimate twice for both inconsistency and imprecision when inconsistency was the cause of imprecision.

## Discussion

In this meta-analysis of 23 studies including 1,039 participants, we found walking to have a large effect on divergent thinking, but no effect on convergent thinking. This review is the first to report meta-analytic evidence of the effects of walking on creative thinking.

The large pooled effect (*d* = 0.93 [95% CI 0.44, 1.42]) of walking on divergent thinking can be considered to be practically meaningful: as a percentile, the average individual, after walking, is likely to outperform over 80% of people with respect to their ability to generate novel ideas [58]. These findings generally align with previous studies on the effect of physical activity and creative ideation [11,23]. As mentioned by previous reviews [11,20], the positive effect is likely driven by both physiological and psychological mechanisms. Aerobic activities, like walking, have been found to improve executive function [59–61] through the increase of cerebral blood flow and upregulation of brain-derived neurotropic factor [61–64], both of which are associated with creativity [65,66]. Psychologically, walking has been shown to improve mood and stress [67–69] which can enhance divergent thinking [34,70]. These physiological and psychological mechanisms may explain the slightly larger effect of outdoor walking (*d* = 1.19 [95% CI 0.09, 2.29]) compared to indoor walking (*d* = 0.92 [95% CI 0.34, 1.50]); outdoor, nature-based exercise has been found to be more effective in improving psychological and physiological outcomes compared to exercise conducted in urban settings or indoors [22,71,72]. Nonetheless, our findings suggest walking, both indoors and outdoors, can both improve the ability to generate creative ideas. These findings could be operationalized in domains that value or require creative ideas, such as educational or professional settings, through activities like walking meetings, which have been found to improve mood and productivity among knowledge-based workers [73] and could be doubly used to stimulate creative idea generation.

We found no evidence of an effect of walking on convergent thinking, which is in line with other studies of physical activity on convergent thinking [23,24]. Relative to divergent thinking, convergent thinking is more closely associated with working memory [74,75], and further, intelligence [76]. Randomized trials have found walking to have limited impact on working memory [77], which may explain the limited effect observed. These results have important implications for the training and cultivation of creative thinking: divergent thinking dominates the creativity literature and is often considered to be synonymous with creative thinking, but convergent thinking plays a critical role in the creative thinking process [18]. Convergent thinking has also been reported to moderate the relationship between divergent thinking and scientific creativity [17]. As such, the ability to effectively exercise convergent thinking to evaluate and select the best ideas generated during the divergent thinking phase is essential – particularly in domains where novel but non-useful ideas have dangerous consequences, like medicine and aviation. While walking may be able to stimulate the generation of creative ideas, our data suggest that other interventions are required to enhance convergent thinking. However, given the lack of studies using a between-subject study design, the limited diversity in convergent thinking assessment tools, and overall uncertainty of the evidence, high-quality randomized trials are required to confirm this result.

We explored several methodological aspects in our study. First, while not statistically significant, we observed a meaningful difference in effect size between between-subject and within-subject study designs: we found a medium-sized effect of walking on divergent thinking in between-subject studies, compared to a very large effect of walking in within-subject studies. It has been noted that between-subject designs tend to be more conservative, while within-subject designs have more statistical power but are susceptible to many confounds including demand and learning effects [78]. Our findings corroborate this claim; the large effect size observed in the primary analysis should be interpreted within this in mind, and future researchers should be aware of the potential implications of the two study design approaches. Second, we found little difference in effect size between divergent thinking assessment methods. While this analysis had limited statistical power – most studies utilized the AUT, with few alternatives – it suggests that most tools of divergent thinking produce reliable results, at least in this context. That being said, the reporting of validity and reliability data was generally poor; future studies would do well to report such data, particularly when novel or uncommon assessment tools are being used. Last, we explored the sensitivity of the effect of walking on divergent thinking by performing an analysis of randomized studies only. We found comparable effect sizes between the primary analysis and this sensitivity analysis, which strengthens our conclusion of a large effect of walking on divergent thinking.

This study has several strengths. First, we conducted a systematic search of multiple databases, conducting all study screening and data extraction independently and in duplicate to minimize the risk of human error or bias. Second, through sub-group and sensitivity analysis, we have explored the differential effect of walking by location, assessment method, and study design, which provides methodological guidance to researchers designing studies of creative thinking and those looking to implement walking interventions for the enhancement of creative thinking. Third, we analyzed divergent thinking and convergent thinking separately, providing meta-analytic data on the effect of walking on all aspects of the cognitive process of creative thinking. Fourth, we performed an assessment of the certainty of the evidence, which is essential in making accurate conclusions and informed decisions based on evidence synthesis results.

### Limitations

This study has some limitations. First, despite multiple sub-group and sensitivity analyses, we were unable to explain much of the heterogeneity observed in the primary meta-analyses. It is possible that other unexplored factors contributed to the unexplained heterogeneity, such as the specifications of the control condition. For example, in the three experiments by Murali and colleagues [48], substantial heterogeneity in the effect estimates were observed (*I*^2^ = 91%), which was hypothesized to be due to lack of explicit instruction of movement restriction in the sitting group in Experiment 1. This exemplifies the potential impact of heterogeneity in the control conditions across studies. Future studies should explore this and other possible moderating factors, such as walking duration and intensity, timing of creativity assessment, educational level, and baseline fitness, that could explain the differential results. Second, while 13 randomized studies were included in the sensitivity analyses of the effect of walking on divergent thinking, many of the positive outcome studies were from a single author (Oppezzo). Given similarities in study design across studies by this author, there is a possible risk of systematic bias; replicative studies are recommended and required to strengthen the supposition of a positive effect. Third, the data on convergent thinking was limited, which reduced the precision of the estimates and precluded any sub-group analyses. Fourth, we chose to use a risk of bias tool that has not been validated and may therefore have methodological limitations. However, this tool was specifically developed for research on physical activity and creativity, and has been successfully used in other peer-reviewed publications [11]: we therefore believe its use is justifiable for this meta-analysis, and more appropriate than conventional tools like the Cochrane Risk of Bias 2.0 or ROBINS-I tools [79,80]. Lastly, most studies were conducted in undergraduate student samples, which limits the generalizability of the results. Future studies in a wider range of populations are necessary to improve the external validity of this evidence.

## Conclusion

In this systematic review and meta-analysis, we found that walking has a large effect on divergent thinking, but no effect on convergent thinking. The results indicate the potential of walking to be used as an intervention for the stimulation of creative idea generation.

## Supporting information

S1 Appendix PRISMA Checklist. (DOCX)

S2 Appendix Search Strategies. (DOCX)

S3 Appendix Study Quality Assessment Tool. (DOCX)

S4 Appendix Study Quality Assessment Table. (DOCX)
