# Supplementary material for: The impact of walking on creative thinking: A systematic review and meta-analysis
Source: PLoS One. 2026 May 13;21(5):e0347878. doi: 10.1371/journal.pone.0347878 (PMC13170883; doi:10.1371/journal.pone.0347878)
Supplement: S2 Appendix — (DOCX) [file pone.0347878.s002.docx]

**Database:**
Ovid MEDLINE(R) ALL <1946 to January 06, 2025>

| **#** | **Query** | **Results from 9 Jan 2025** |
| --- | --- | --- |
| 1 | (bodily movement or walk* or treadmill).ti,ab. | 188,677 |
| 2 | (creativ* or creative thinking or divergent thinking or convergent thinking or creative potential open problem solving or creative ideation or originality or fluency or remote associates or Torrance or alternate uses or AUT or TTCT).mp. | 54,153 |
| 3 | creativity/ or divergent thinking/ | 8,159 |
| 4 | exp Walking/ | 72,438 |
| 5 | 1 or 4 | 214,239 |
| 6 | 2 or 3 | 54,153 |
| 7 | 5 and 6 | 513 |

(bodily movement or walk* or treadmill).ti,ab.
(creativ* or creative thinking or divergent thinking or convergent thinking or creative potential open problem solving or creative ideation or originality or fluency or remote associates or Torrance or alternate uses or AUT or TTCT).mp.
creativity/ or divergent thinking/
exp Walking/
1 or 4
2 or 3
5 and 6


<https://libaccess.mcmaster.ca/login?url=http://ovidsp.ovid.com/ovidweb.cgi?T=JS&NEWS=N&PAGE=main&SHAREDSEARCHID=5NAiZNHVHg5nJynBFqyVRVo4CB63UPvzsH0AVcD8sqHXa2p29qK30Vj4uOThRr55q>

**Database:**
APA PsycInfo <1806 to December 2024 Week 5>

| **#** | **Query** | **Results from 9 Jan 2025** |
| --- | --- | --- |
| 1 | (bodily movement or walk* or treadmill).ti,ab. | 35,844 |
| 2 | (creativ* or creative thinking or divergent thinking or convergent thinking or creative potential open problem solving or creative ideation or originality or fluency or remote associates or Torrance or alternate uses or AUT or TTCT or RAT).mp. | 207,345 |
| 3 | exp Female Animals/ or exp Animals/ or exp Service Animals/ or exp Male Animals/ | 387,766 |
| 4 | creativity/ or divergent thinking/ | 31,963 |
| 5 | exp Walking/ | 7,826 |
| 6 | 1 or 5 | 36,431 |
| 7 | 2 or 4 | 207,345 |
| 8 | 6 and 7 | 1,292 |
| 9 | 8 not 3 | 715 |

(bodily movement or walk* or treadmill).ti,ab.
(creativ* or creative thinking or divergent thinking or convergent thinking or creative potential open problem solving or creative ideation or originality or fluency or remote associates or Torrance or alternate uses or AUT or TTCT or RAT).mp.
exp Female Animals/ or exp Animals/ or exp Service Animals/ or exp Male Animals/
creativity/ or divergent thinking/
exp Walking/
1 or 5
2 or 4
6 and 7
8 not 3


<https://libaccess.mcmaster.ca/login?url=http://ovidsp.ovid.com/ovidweb.cgi?T=JS&NEWS=N&PAGE=main&SHAREDSEARCHID=6D1K2UySzxGJCmXbz2hRusqoZNKwedmvszMVpHxd9s3trvPjSWCkrcZ4zL3Hgjcr3>

**Scopus and ProQuest:**

( "bodily movement" OR walk* OR treadmill ) AND ( creativ* OR "creative thinking" OR "divergent thinking" OR "convergent thinking" OR "creative potential" OR "open problem solving" OR "creative ideation" OR originality OR fluency OR "remote associates" OR torrance OR "alternate uses" OR aut OR ttct)
