# Supplementary material for: The impact of walking on creative thinking: A systematic review and meta-analysis
Source: PLoS One. 2026 May 13;21(5):e0347878. doi: 10.1371/journal.pone.0347878 (PMC13170883; doi:10.1371/journal.pone.0347878)
Supplement: S3 Appendix — (DOCX) [file pone.0347878.s003.docx]

**Appendix A2. Study quality assessment protocol from Frith and colleagues.**

| Item | Longitudinal study | Rating |
| --- | --- | --- |
| (1) | Was the physical activity manipulation controlled (e.g., completed in a laboratory setting, standardized by duration and intensity, and for interaction with other participants if administered in a group context)?*   - RCTs and pre-post get 1, cross-sectional 0 | Yes (1)/No (0) |
| (2) | Was there evidence of validity for the creativity measure(s) utilized? | Yes (1)/No (0) |
| (3) | Was there evidence of reliability for the creativity measure(s) utilized? | Yes (1)/No (0) |
| (4) | Were creativity scoring and evaluation procedures robust to bias (e.g., blinded scoring completed by multiple researchers, provision of strong interrater reliability, and detailed or referenced?   - Multiple reviewers (with or without blinding, with or without agreement statistics) or AI scoring | Yes (1)/No (0) |
| (5) | Were random group assignment and/or counterbalancing procedures appropriate (e.g., were participants assigned to groups based on course enrollment, rather than random selection and were the order of creativity assessments randomized to ensure resistance to temporal artifacts or learning effects?) for the study design?   - Only RCTs get 1 | Yes (1)/No (0) |
| (6) | Did the intervention use a non-exercise control group or condition? *   - RCTs and pre-post get 1 | Yes (1)/No (0) |
| (7) | Were statistically appropriate/acceptable methods of data analysis used? | Yes (1)/No (0) |
| (8) | Were point estimates, standard deviations, confidence intervals, and/or effect sizes reported? | Yes (1)/No (0) |
| Total Score | | / 8 |

Frith, E., Ryu, S., Kang, M., Loprinzi, P.D., 2019. Systematic review of the proposed associations between physical exercise and creative thinking. Europe’s Journal of Psychology 15, 858–877.. https://doi.org/10.5964/ejop.v15i4.1773
