# Supplementary material for: The impact of walking on creative thinking: A systematic review and meta-analysis
Source: PLoS One. 2026 May 13;21(5):e0347878. doi: 10.1371/journal.pone.0347878 (PMC13170883; doi:10.1371/journal.pone.0347878)
Supplement: S4 Appendix — (DOCX) [file pone.0347878.s004.docx]

| **Risk of Bias Item** | | | | | | | | | | |
| --- | --- | --- | --- | --- | --- | --- | --- | --- | --- | --- |
| **Study** | **1** | **2** | **3** | **4** | **5** | **6** | **7** | **8** | **Total** | **Classification** |
| Between-Subject Designs | | | | | | | | | | |
| Bartholomae 2024 | X | X | X | X | X | X |  | X | 7/8 | Low |
| Leung 2012 (Study 2b) | X |  |  | X | X | X |  | X | 5/8 | Moderate |
| Main 2018 (Experiment 1) | X | X | X | X | X | X |  | X | 7/8 | Low |
| Main 2018 (Experiment 2) | X | X | X | X | X | X | X | X | 8/8 | Low |
| Oppezzo 2012 (Experiment 1) | X | X | X |  | X | X | X | X | 7/8 | Low |
| Oppezzo 2012 (Experiment 2) | X |  |  | X | X | X | X | X | 6/8 | Low |
| Oppezzo 2012 (Experiment 3) | X | X | X |  | X | X | X | X | 7/8 | Low |
| Oppezzo 2014 (Experiment 2) | X | X | X | X | X | X | X | X | 8/8 | Low |
| Oppezzo 2014 (Experiment 3) | X | X | X | X |  | X | X | X | 7/8 | Low |
| Palmer 1995 | X | X | X |  | X | X | X | X | 7/8 | Low |
| **Within-Subject Designs** | | | | | | | | | | |
| Abdullah 2016 | X | X | X | X |  | X | X | X | 7/8 | Low |
| Frith 2018 | X | X | X |  | X | X | X | X | 7/8 | Low |
| Frith 2021 | X |  |  | X | X | X | X | X | 6/8 | Low |
| Frith 2022 | X | X | X | X | X | X | X | X | 8/8 | Low |
| Jung 2023 | X |  |  | X | X | X | X | X | 6/8 | Low |
| Murali 2022 (Experiment 1) | X | X | X |  | X | X | X | X | 7/8 | Low |
| Murali 2022 (Experiment 2) | X | X | X |  | X | X | X | X | 7/8 | Low |
| Murali 2022 (Experiment 3) | X | X | X |  | X | X | X | X | 7/8 | Low |
| Oppezzo 2014 (Experiment 1) | X | X | X | X |  | X | X | X | 7/8 | Low |
| Patterson 2018 | X | X | X | X |  | X | X | X | 7/8 | Low |
| Zhou 2017 (Experiment 1B) | X |  |  | X | X | X | X | X | 6/8 | Low |
| **Cross-Sectional** | | | | | | | | | | |
| Rominger 2024 |  | X | X | X |  |  | X |  | 4/8 | Moderate |
| Cheng 2021 |  |  |  | X |  |  | X | X | 3/8 | Moderate |
